# Supplementary material for: Two-photon absorption under few-photon irradiation for optical nanoprinting
Source: Nat Commun. 2025 Mar 1;16:2086. doi: 10.1038/s41467-025-57390-9 (PMC11871329; doi:10.1038/s41467-025-57390-9)
Supplement: Supplementary file 1 — Supplementary Information [file 41467_2025_57390_MOESM1_ESM.pdf]

Supplementary Materials for

Two-photon absorption under few-photon irradiation for optical nanoprinting

Zi-Xin Liang[1†], Yuan-Yuan Zhao[1†*], Jing-Tao Chen[1†], Xian-Zi Dong[2†], Feng Jin[2], Mei-Ling Zheng[2*], Xuan-Ming Duan[1*]

[1] Guangdong Provincial Key Laboratory of Optical Fiber Sensing and Communications, Institute of Photonics Technology, Jinan University, Guangzhou 510632, P. R. China

[2] Laboratory of Organic NanoPhotonics and CAS Key Laboratory of Bio-Inspired Materials and Interfacial Science, Technical Institute of Physics and Chemistry, Chinese Academy of Sciences, Beijing 100190, P. R. China

* Corresponding author. Email: xmduan@jnu.edu.cn, zhengmeiling@mail.ipc.ac.cn, yyzhao@jnu.edu.cn

† These authors contributed equally to this work.

This PDF file includes:

Supplementary Notes (Notes 1-19)

Supplementary information figures (Suppl. Fig.1 - Suppl. Fig.17)

Supplementary information tables (Suppl. Table 1 - Suppl. Table 4)

Supplementary Reference

Supplementary Note 1: Simulation modeling

According to the characteristics of the DMD, the illumination source of the digital projection optical system in this study is described as parallel light illumination emitted by a point light source. The vector imaging model rather than the traditional scalar model at the larger numerical aperture (N.A.) is considered. The point spread function (PSF) is convolved with the complex amplitude transmittance function of the DMD after loading the digital mask to obtain the light intensity distribution in the macro space. According to the experimental results, we must consider the impact of lifetime on two-photon absorption under a few photons according to the quantum theory. The Monte Carlo random algorithm is used to obtain the random distribution of the number of photons in a single pulse based on the intensity distribution (*1*). The number of photons in the spatial position is also randomly distributed according to the intensity, and the photon number distribution images in space and time are obtained, respectively. MATLAB is then used to statistically screen the number of photons and obtain an effective logarithmic distribution of two-photon reactions based on the space-time conditions met by two-photon absorption.

Supplementary Note 2: Modeling of the light intensity distribution during vector imaging model

We define the local coordinate system (*s, p*) at the same time considering a parallel light ray traveling along a wave vector $\hat{\boldsymbol{k}} = [\alpha, \beta, \gamma]$. The coordinate axes in the local coordinate system change with the direction of the wave vector k of the light wave. The s-axis is always perpendicular to the plane formed by the wave vector *k* and the *z*-axis. The *p*-axis is in the plane formed by the wave vector $\hat{\boldsymbol{k}}$ and the *z*-axis. At the same time, it is orthogonal to the *s*-axis. For a parallel light whose polarization component is described using the (*s, p*) coordinate system, its polarization component in the global coordinate system (*x, y, z*) can be calculated according to the wave vector $\hat{\boldsymbol{k}}$ of its light wave, as shown in Suppl. Fig.1. α is the cosine value of the angle between the wave vector $\hat{\boldsymbol{k}}$ and the *x*-axis, β is the cosine value of the angle between the wave vector $\hat{\boldsymbol{k}}$ and the *y*-axis, γ is the cosine value of the angle between the wave vector $\hat{\boldsymbol{k}}$ and the *z*-axis, and satisfies the relationship $\alpha^2 + \beta^2 + \gamma^2 = 1$.

$$\begin{bmatrix} E_x \\ E_y \end{bmatrix} = T \begin{bmatrix} E_s \\ E_p \end{bmatrix} \quad (1)$$

Where *T* represents the transformation of the (*s, p*) coordinate system to the (*x, y*) coordinate system.

Initially consider that a point source in the illumination pupil coordinate system (α_s, β_s) emits parallel light. In the context of the thin mask approximation, the diffraction field in the near field of the mask is as follows:

$$E_{mask}(a,\beta) = m(a - a_s, \beta - \beta_s)T(\alpha_s,\beta_s)\begin{bmatrix} Es \\ Ep \end{bmatrix} \tag{2}$$

Where *m* is the complex amplitude transmittance of the digital mask loaded with DMD. According to the characteristics of the digital mask that can be loaded on the DMD digital micromirror:

$$m(x,y) = \sum_i \sum_j M_{i,j} \times \text{rect}\left(\frac{x-i\cdot P}{w}, \frac{y-j\cdot P}{w}\right) \tag{3}$$

$M_{i,j} = 1$ "on" state

$M_{i,j} = 0$ "off" state

Among them, *i* and *j* represent the rows and columns of the DMD respectively; $M_{i,j}$ represents the modulation function loaded on a certain row and column of the DMD (the phase difference is not considered in the DMD system, so only normalization is considered). The amplitude is 1 in the on state or 0 in the off state. *w* represents the DMD pixel's size while *P* is the DMD pixel's period. In this study, we do not consider the duty cycle of the DMD pixel, that is, *P* = *w* = 7.56 μm.

Then, we can get the polarized light field calculated from the object plane mask to the image plane where the sample is located:

$$\begin{bmatrix} E_x^{wafer}(\alpha',\beta') \\ E_y^{wafer}(\alpha',\beta') \\ E_z^{wafer}(\alpha',\beta') \end{bmatrix} = \begin{bmatrix} H_x(\alpha',\beta') \\ H_y(\alpha',\beta') \\ H_z(\alpha',\beta') \end{bmatrix} m(\alpha - \alpha s, \beta - \beta s) \tag{4}$$

$H(\alpha',\beta')$ is the frequency response function of the vector projection optical system considering the point light source [2]:

$$\begin{bmatrix} H_x(\alpha',\beta') \\ H_y(\alpha',\beta') \\ H_z(\alpha',\beta') \end{bmatrix} = R(\gamma,\gamma')\cdot C(\alpha',\beta')B(\alpha',\beta')T^{-1}(\alpha,\beta)T(\alpha s,\beta s)\begin{bmatrix} Es \\ Ep \end{bmatrix} \tag{5}$$

$$T^{-1}(\alpha,\ \beta) = \frac{1}{\gamma}\begin{bmatrix} -\frac{\beta\gamma}{\rho} & \frac{\alpha\gamma}{\rho} \\ -\frac{\alpha}{\rho} & -\frac{\beta}{\rho} \end{bmatrix} \tag{6}$$

$$B(\alpha',\beta') = \begin{bmatrix} \frac{-\beta'}{\rho'} & -\frac{\alpha'\gamma'}{\rho'} \\ \frac{\alpha'}{\rho'} & \frac{-\beta'\gamma'}{\rho'} \\ 0 & \rho' \end{bmatrix} \tag{7}$$

We transform the light field described in the frequency domain back to the spatial domain through Fourier transformation to obtain the PSF:

$$\begin{bmatrix} h_x(x',y') \\ h_y(x',y') \\ h_z(x',y') \end{bmatrix} = \iint \begin{bmatrix} H_x(\alpha',\beta') \\ H_y(\alpha',\beta') \\ H_z(\alpha',\beta') \end{bmatrix} \times e^{j2\pi(f'x'+g'y')} \mathrm{d}\alpha' \mathrm{d}\beta' \tag{8}$$

After the derivation, for convenience, (x, y) represents the image plane where the photoresist is located, and $m(x, y)$ represents the ideal image of the digital mask passing through the projection system. Then we consider the vector model in the spatial calculation, add the point spread function $h_p(x, y)$ of a certain polarization direction and the complex amplitude transmittance of the DMD for convolution calculation, and then take the square of its absolute value. Finally, the light intensity distribution function after loading the DMD digital mask was obtained. h_p represents the PSF in a certain polarization direction, * is the convolution operation, and I is the projected light intensity distribution.

$$I(x, y) = \sum_{p=x,y,z} |m(x, y) * h_p(x, y)|^2 \tag{9}$$

Supplementary Note 3: Modeling of photon distribution in quantum models

We transition from the light intensity distribution of the semi-classical theory to the fully quantum theory when the dose is gradually reduced. When the macroscopic light field is transferred to the quantized photon distribution, it is inevitable to obtain the photon distribution. Based on the necessary conditions for the occurrence of two-photon absorption, the same molecule must absorb pairs of photons with combined energy exceeding the lowest excited level energy within the lifetime of the virtual state. There are three states for an active molecule in space, namely the initial state, the intermediate state, and the excited state. The area can no longer absorb the photon in the excited state. The active molecule in the initial state will transit after absorbing one photon. To reach the intermediate state, the active molecule absorbs another photon and jumps to the excited state. Recording the binding of a photon pair within the lifetime of the virtual state is identified as a potential instance of TPA. Within its lifetime, it will return to the initial state and will not be counted as part of the *e*TPA number if the active molecule in the intermediate state does not receive other photons.

(1) Spatial distribution of photon number within a single pulse:

The spatial probability density function $P_S(x, y)$ is obtained by normalizing the light intensity. For a specified two-dimensional area, the probability of a photon appearing under this light intensity is $I(x, y)$.

$$P_S(x, y) = \frac{I(x,y)}{\iint_{mask} I(x_i, y_j) dxdy} \tag{10}$$

$$\int_0^x \int_0^y P_S(x, y) dydx = 1 \tag{11}$$

Given the experimental dose (single pulse single pixel photon number, N_{spp}), a random spatial distribution of the single pulse spatial photon number in position (x, y) is obtained.

$$n(x, y) = N_{spp} * P_S(x, y) \tag{12}$$

(2) Distribution of photon number in time within a single pulse:

The distribution of pulses in time fits the sech function:

$$U(t) = \exp(-iw\,t)\text{sech}\left(\frac{t}{\Gamma}\right) \tag{13}$$

The center frequency is w, t is the local time coordinate. Γ represents the pulse width when the amplitude drops to $1/e$ of the peak value and it is a measurement of the pulse time width.

The time function $P_T(t)$ represents the probability density distribution function of the number of photons in a single pulse in time, and the integral within the time of a single pulse is 1:

$$P_T(t) = \frac{U(t)}{\iint U(t)dt} \tag{14}$$

$$\int_0^t P_T(t)dt = 1 \tag{15}$$

Given the experimental dose (single pulse single pixel photon number, N_{spp}), a random time distribution of the single pulse spatial photon number is obtained.

$$n(t) = N_{spp} * P_T(t) \tag{16}$$

The random distribution of photon numbers within a single pulse is shown in Suppl. Fig.2 (a pulse width of ~238 fs).

(3) Enhanced spatiotemporal distribution with added lifetime:

Quantifying TPA events at a single point: (r, t_n) represents a photon sample at a certain position *r* at a certain time *t*. The spatial distribution (r) of the photon conforms to the distribution $r \sim P_S(r)$, while the temporal distribution (t) conforms to the distribution $t \sim P_T(t)$. The state function at this point and moment is represented by $S(r, t_n)$. When it is 1, it means that two-photon absorption will occur at this point and that moment to produce active molecules. When it is 0, it means that this point and that moment are in a virtual state. It is assumed here that there are no two photons at exactly the same time and in the same place.

$$S(r, t_{n+1}) = \begin{Bmatrix} 1 & t_{n+1} - t_n < \tau \quad and \quad S(r, t_n) = 0 \\ 0 & otherwise \end{Bmatrix} \tag{17}$$

If the photons corresponding to the same point (r, t_{n+1}) and the same point at the previous moment (r, t_n) meet the specified conditions, two-photon absorption will occur. According to the

Heisenberg Uncertainty Principle, the intrinsic lifetime of virtual state satisfies the following equation:

$$\tau_i = h(4\pi \Delta \tilde{v}_{i\lambda})^{-1} \tag{18}$$

Where h is Planck's constant and $\Delta \tilde{v}_{i\lambda}$ is the difference between photon energy and inital state energy. The photoresist exhibits a cutoff wavelength of 353 nm. Utilizing a laser beam with a wavelength of 517 nm, the energy differential between the first excited state and the inital state is quantified at 1.1 eV, resulting in a calculated virtual state lifetime of 0.3 fs. Furthermore, the lifetime of the virtual state extends to 0.8 fs when exposed to a laser with a wavelength of 400 nm.

Finally, the distribution of two-photon absorption pairs, where TPA occurs within a certain point, is calculated as follows:

$$TPA(r) = \sum_{n=0}^{N} S(r, t_n) \tag{19}$$

Although we cannot calculate its specific analytical formula, the distribution of *e*TPA can be obtained by performing the above calculation based on the number of input pulse photons. According to our statistics, the TPA distribution in a certain situation, in the case of few photons: $TPA(r) \in I^2$, the distribution of TPA is proportional to the square of the light intensity. The probability of a photon falling is expected to be the distribution of light intensity, and the probability of a two-photon reaction is expected to be the distribution of the square of the light intensity.

$$E_{Nphoton} = norm(I) \tag{20}$$

$$E_{reaction} = norm(I^2) \tag{21}$$

(4) Distribution of reaction site number in time within a single pulse:

The reaction site number *R* of the photosensitive molecule in the photoresist is consistent with the Dill model (*33,34*):

$$\frac{\partial[R]}{\partial t} = -C_{Dill} \cdot I \cdot [R] \tag{22}$$

Where *C* is the effective rate constant for the photoreaction and the third Dill photoresist parameter. Subsequently, the concentration distribution of *R* is obtained following the integration of R's first-order kinetics concerning to incident light intensity:

$$[R] = -e^{-C_{Dill} \cdot I \cdot t} = -e^{-C_{Dill} \cdot D} \tag{23}$$

Where *t* and *D* are the exposure time and dose, respectively. We gain a detailed understanding of how the concentration of the *R* responds to varying levels of incident light by linking the concentration distribution to the previously mentioned incident dose. The actual concentration distribution *H* of *R* participating in the reaction is expressed as formula 24, with the initial *R*

concentration (R_0) set to a value greater than or equal to the previously calculated maximum value of N_{eTPA} (as indicated in the main text with R_0=40) at t=0.

$$[H] = [R]_0\left(1 - e^{-C_{Dill}\cdot[TPA]}\right) \tag{24}$$

This correlation provides essential insights into the direct relationship between R concentration and incident light dose, crucial for optimizing processes in applications such as photolithography. In our calculations, we also considered other influencing factors, such as the refractive index of the photoresist. However, since the absorptivity of the photoresist depends on various factors, including its concentration and thickness, the actual effect may vary with different resists. To minimize the impact of the Z-direction height on the effective reaction distribution, we have opted to reduce the film thickness in our experiments. For the theoretical calculations, we have temporarily excluded the specific value of absorptivity in this study. The relevant parameters are calculated and summarized in Suppl. Table 1.

Supplementary Note 4: The distribution of *e*TPA at different wavelengths

Taking N_{spp} = 6000 (2.31 fJ/(pulse ·pixel)) and N_{pulse} = 700 as typical cases, the distribution of *e*TPA at wavelengths of 517 nm and 400 nm and pulse widths of 238 fs and 100 fs were simulated respectively. Given that the photon energy of 400 nm is relatively greater than that of 517 nm (E_{517} = 2.4 ev, E_{400} = 3.1 ev), we additionally computed the distribution at 400 nm with N_{spp} set to 4645 (1.78 fJ/(pulse·pixel)) (Suppl. Fig.3 e-f) under the condition where the total energy of 517 nm and 400 nm is equal. We make statistics on the number of *e*TPA in different situations, as shown in Suppl. Fig.3 and Suppl. Table 2. The efficiency is the ratio of the number of incident photons to *e*TPA.

Supplementary Note 5: Coefficient of variance of TPA occurrence probability under different photon numbers injected

In our random calculation results, the position where photons fall and react is not fixed. Here we show the results of ten random calculations of TPA reaction under three levels of incident photon numbers. The incident photon numbers are as follows: (a) N_{spp} = 600 (0.23 fJ/pulse pixel), (b) N_{spp} = 6000 (2.31 fJ/(pulse·pixel)), (c) N_{spp} = 30000 (11.53 fJ/pulse pixel), with a total pulse number of the pulse is 700 (Suppl. Fig.4). The random results are different under the same incident conditions. The probability distribution of the response at a specific moment cannot be determined experimentally, yet the origin of the minimum feature size can be identified. The random fluctuation is more obvious under a few numbers of photons. Due to the varying magnitudes of injected photons, there is a significant difference in the mean values. Therefore, we use the coefficient of variation C_V (standard deviation/mean value) to eliminate the influence of different data sets on the degree of dispersion. The

degree of dispersion of the entire spot is 9.79 %, 0.82 %, and 0.15 %, respectively, as shown in Suppl. Table 3.

$$C_V = \frac{\sigma}{\mu} \times 100\% \quad (25)$$

Supplementary Note 6: Effect of pulse number on *fp*TPA

The efficiency of two-photon absorption is limited by the virtual state. The occurrence efficiency f_{TPA} is only related to the number of photons in a single pulse under a single pixel and has nothing to do with the number of pulses. Taking 517 nm as an example, the number of photons in a single pixel of Suppl. Fig.5 a is 514, and the number of pulses is 5×10^4, 1×10^5, 2×10^5, and 4×10^5 in a proportional increasing relationship. Suppl. Fig.5 a shows the photon distribution of 1/4 of the light spot, Suppl. Fig.5 b is the photon distribution of 1/4 spot under the proportional increasing relationship of 5.14×10^6 photons in a single pulse and single pixel, and the number of pulses is 5, 10, 20, and 40, respectively. Suppl. Fig.5 c represents the distribution cross-section of a-4 and b-4, which only aligns with the distribution of the square of the light intensity when the number of photons is low. Suppl. Fig.5 d shows the distribution of $N_{e\mathrm{TPA}}$ at 517 nm and 400 nm when the number of pulses increases from 1 to 1500 when N_{spp} = 6000 (2.31 fJ/(pulse·pixel)) and N_{spp} = 10000 (3.84 fJ/pulse pixel), indicating that the increase in the number of pulses only results in a linear increase in $N_{e\mathrm{TPA}}$ without altering its occurrence efficiency.

Supplementary Note 7: Computational model in MATLAB

The computational model was established in MATLAB by numerically representing equations 1-24. The optical setup uses on-axis point light source illumination with a polarization direction of x-direction and a wavelength of 517 nm. The numerical aperture (NA) of the projection imaging system is 1.49, and the refractive index in object space (such as the plane where the DMD is located) and image space (such as the plane where the photoresist is located) are 1.0 and 1.516, respectively. Subsequently, an optical projection system reduces the size of the image by a factor of 100 and projects it onto a silicon wafer. The size of the DMD pixel is 7.56 μm. Considering 100% filling of DMD pixels, the complex amplitude transmittance of each DMD pixel can be discretized into a 20×20 matrix with all “1”, indicating that the smallest unit on the sampling interval of the image plane where the silicon wafer is located is set to 3.78 nm. The effects of various optical parameters on the distribution of the effective two-photon absorption number are illustrated in Suppl. Fig.6.

Supplementary Note 8: Optical setup of two-photon optical projection lithography

Femtosecond laser pulses with a fundamental wavelength of 1035 nm are frequency-doubled to

517 nm by a fiber laser (CAS Microstar; pulse width: 238 fs, repetition rate: 1 MHz) for all subsequent two-photon polymerization (TPP) fabrication experiments. The power is adjusted using an electro-optical attenuator (Jie Dong Precision, China). To fully utilize the DMD (DLP6500, 1920 × 1080 pixels, 7.56 µm pixel size, 0.65-inch diagonal micromirror array, optimized for visible light (420 nm–700 nm), projection frequency 1-22.7 kHz, Texas Instruments, a 1× - 3× zoom beam expander (BEZHP-1/3-500/570, Sigmakoki) is employed to expand the laser spot diameter to 6 mm. This allows the output light from the πShaper 6_6_VIS (Adloptica) to pass through the beam expander and produce a top-hat beam with a diameter of 18 mm, without changing the irradiance *(2)*. The maximum diffraction efficiency is achieved by optimizing the incident angle (θ_{in}) to 34°, and the effective energy utilization (η_{eff}) can reach up to 88%, as shown in Suppl. Fig.7b (*3*). The optical system can project any binarized pattern loaded onto the DMD onto the target photoresist through the tube mirror (TTL200-UVB, Thorlabs) and the objective lens (Apo TIRF, oil, 100×/1.49 NA, Nikon) without moving the stage (Suppl. Fig.7a).

Supplementary Note 9: Photopolymerization

The two-component compound of the non-chemically amplified negative photoresist (AR-N 7520) used in the experiment consists of a bisazide compound and a polyhydroxy phenolic compound. It absorbs two-photon simultaneously and is excited to an excited state by laser irradiation. This excitation can only trigger an insertion reaction between the azide group of the bisazide compound, serving as a cross-linking agent, and the alkyl group of the phenolic compound. This reaction forms dimers or polymers, increasing the molecular weight and the polymerized polymers become insoluble in the developer. UV-Vis absorption spectrum shows that there are two absorption peaks at 323 nm and 284 nm in Suppl. Fig.8. The single-photon absorption cutoff wavelength is 353 nm, which indicates that a single-photon absorption reaction can hardly occur at 517 nm (Abs $_{(400)}$ = 0.0411, Abs $_{(517)}$ = 0.0067), thus paving the way for the occurrence of two-photon absorption.

Supplementary Note 10: Estimation of fabrication rate

"Throughput" is an important parameter for characterizing lithography efficiency, typically representing the area or volume of patterned structures produced per unit of time. The definition of throughput slightly varies across different lithography technologies, for example:

(1) In traditional mask-based lithography, "throughput" is generally defined as the number of wafers processed per hour, with units in wafers per hour (WPH) (*4*). For example, an EUV lithography machine using 12-inch wafers (300 mm) has a throughput of 170 WPH.

(2) For 2D patterning, in processes such as electron beam lithography, laser direct-write lithography, or maskless UV lithography (*5*), “throughput” is defined as the area exposed per minute, with units in mm^2/min or mm^2/s.

(3) For 3D patterning, in two-photon lithography (*6*), since the smallest exposure unit is a voxel, “throughput” is defined as the number of voxels exposed per second, with units in Voxels/s. Once the voxel size is determined, it can be converted into mm^2/min or mm^2/s. In UV-cured 3D printing (*7*), “throughput” is defined as the volume exposed per second, with units in mm^3/s.

In our research, we focus on 2D patterning of photoresists, where “throughput” is defined as the area exposed per minute, with units in mm^2/min. The πShaper is employed for homogenization and achieves a high transmittance of 98%, thereby minimizing energy loss. The consistency of the laser beam does not deteriorate and there is no focus inside from the homogenized spot in Suppl. Fig.9 a–b. The uniformity of the entire light field is authenticated using a photon sieve pattern with a large diameter of 80 μm ($N_{pulse} = 1 \times 10^7$ and $N_{spp} = 2.38 \times 10^4$ (9.14 fJ/(pulse·pixel))) and a resolution plate pattern with a length of 100 μm ($N_{pulse} = 8 \times 10^6$ and $N_{spp} = 3.51 \times 10^4$ (13.49 fJ/pulse pixel)). Large-scale pattern exposure at the scale of 100 microns can be achieved in a single exposure under the two-photon optical system, achieving a fabrication rate of 1×10^{-3} mm^2/s (Suppl. Fig.9 c-f). Under exposure with the DLP 9000 (2560 × 1600 pixels, 7.56 μm pixel pitch, Texas Instruments) and 40x objective lens (Fluar, 1.30 Oil, Zeiss), a single exposure area of 250 μm × 400 μm can be achieved, realizing a fabrication rate of 0.1 mm^2/s.

Supplementary Note 11: Detailed Analysis of N_{eTPA} Distributions corresponding to the experimental data

Suppl. Fig.10 corresponds to the experimental results of Fig 3e in the main text. Suppl. Fig.10 a shows the total distribution of *eTPA*, with a uniform color bar ranging from 0 to 1000. Suppl. Fig.10 b displays the distribution after extracting the contour of the linewidth, indicating the distribution where N_{eTPA} exceeds a certain value within a single sampling point, corresponding to the exposure linewidth. The inset shows the distribution of N_{eTPA} at the minimum feature size.

Supplementary Note 12: Feature dimensions line edge roughness

Apply an edge extraction algorithm to extract data from the entire polymer line in the SEM, followed by the calculation of edge roughness σ_{LER}. The edge roughness of the feature size due to the influence of quantum noise is shown in Suppl. Fig.11. The edge roughness (LER) is usually described quantitatively by using 3 times the edge standard error (ie, $3\sigma_{LER}$) in the field of lithography.

$$\sigma_{LER} = \sqrt{\frac{1}{N}\sum_{i=1}^{N}(\delta x_i)^2} = \sqrt{\frac{1}{N}\sum_{i=1}^{N}(x_i - \bar{x})^2} \tag{25}$$

For the top edge, $\sigma_{LER(a)} = 4\ \mathrm{nm}$, $\sigma_{LER(b)} = 3\ \mathrm{nm}$; for the bottom edge, $\sigma_{LER(a)} = 4\ \mathrm{nm}$, $\sigma_{LER(b)} = 4\ \mathrm{nm}$. The standard errors for the difference in the edge extraction data (the line width) and the line width roughness are calculated as 5 nm and 7 nm, respectively.

Supplementary Note 13: Sparrow criterion for parallel exposure

In parallel lithography, the Sparrow criterion is analogous to the resolution principles of traditional projection lithography employing physical masks (*8,9*). In projection imaging systems, the Sparrow criterion is established based on the condition that the optical system's modulation transfer function (MTF) equals zero (*10,11*). When the line periodic resolution reaches the value defined by the Sparrow criterion, the corresponding MTF condition allows for a resolvable image in the photoresist (*11*). Under axial point source illumination, the cutoff frequency of the projection optical system is defined as NA/λ (where the +1 or -1 diffraction orders are situated at the edge of the entrance pupil), leading to a theoretical resolution of d=λ/NA for lithographic imaging (*12*).

For single-photon exposure, the coherent linear superposition of light intensity from parallel exposure results in a local minimum appearing precisely at the center, with the exposure dose distribution of the adjacent projection light fields (proportional to intensity *I*) being resolvable, as shown in Suppl. Fig.12(b, d). Even with two-photon exposure, the resolvable pitch for the exposure dose between adjacent projection light fields remains at the critical value of d=λ/NA, constrained by the minimum spacing resulting from the superposition of adjacent light intensities; the exposure dose is simply proportional to I^2. Thus, d=λ/NA represents "the general Sparrow criterion" for parallel projection lithography.

When employing off-axis point source illumination, such as through oblique illumination (*9,13*) or equivalent phase-shifting masks (*9,14*), the cutoff frequency of the projection optical system increases to 2NA/λ (which shifts the diffraction spectrum to position the + 2 diffraction order at the edge of the entrance pupil), doubling the theoretical resolution to d=λ/(2NA). Regardless of whether single-photon or two-photon exposure is utilized, the resolvable pitch for the exposure dose of adjacent projection light fields remains at the critical value of d=λ/(2NA), again limited by the minimum spacing from adjacent light intensity superposition, as depicted in Suppl. Fig.12(c, e). Consequently, d=λ/(2NA) defines the Sparrow criterion for parallel projection lithography under off-axis illumination conditions.

The digital mask projection lithography system discussed in this manuscript uses a collimated ultrafast laser beam aligned parallel to the optical axis, equivalent to the illumination of an object point at infinity on-axis. This setup corresponds to "the general Sparrow criterion" for parallel projection lithography, where d=λ/NA. In our experiments, we used a laser with a center wavelength of 517 nm

and an objective lens with an NA of 1.49, yielding a theoretical line pitch resolution of approximately 347 nm. Thus, a single exposure cannot achieve a line array pattern with period of 227 nm (corresponding to 3 pixels). However, by using double exposure (with a single-exposure pattern resolution of 454 nm, equivalent to 6 pixels, which adheres to the Sparrow criterion), we obtained clear, distinguishable exposure results, as shown in Fig. 4 of the manuscript.

Supplementary Note 14: Raster double exposure detail

The 2:1 periodic lines exhibit misalignment and splitting, while the 2:4 periodic grating pattern is employed for exposure. Suppl. Fig.13 illustrates an otherwise indistinguishable single exposure result when exposing the layout at the same exposure dose.

Supplementary Note 15: The effect of line spacing on exposure dose perception

The spacing between lines directly influences the degree of reaction between adjacent lines, and the required exposure dose varies for lines with different densities. A comprehensive analysis was conducted on the calculation of optical field intensity between sets of lines, each consisting of a 1-pixel width, where the line spacing gradually increased from 1 pixel to 15 pixels. The optical field intensity experienced by lines at different positions undergoes variations due to interference and diffraction effects between adjacent lines when the spacing is less than 5 pixels. For instance, only two out of the five lines are distinguishable with a 1-pixel spacing, regardless of dosage control. Four lines become discernible at 2-pixel spacing. It’s only when the spacing reaches 3 pixels or more that all five lines can be fully displayed, albeit with a non-uniform intensity distribution. The influence of adjacent lines becomes negligible when the spacing has increased to 5 pixels or beyond, as illustrated in Suppl. Fig.14.

Supplementary Note 16: Exposure details of chip metal layer

For the basic circuit layout of the metal layer of the chip, a double in-situ exposure is carried out after splitting. Among them, the three regions of primary interest containing a 1-pixel interval are specifically indicated with gray and white squares (Suppl. Fig.15 a). As demonstrated in the experimental SEM image (Suppl. Fig.15 b), the minimum gap of 37 nm can be obtained under the conditions of N_{spp} = 15200 (2.31 fJ/(pulse·pixel)), $N_{pulse} = 1.5 \times 10^7$ ($T_{MASK1} = T_{MASK2}$ = 15 s).

Supplementary Note 17: Details of a microring waveguide

The number of pulses is increased from $1{\times}10^6$ to $2.1{\times}10^7$ while N_{spp} is fixed ($N_{spp} = 1.49{\times}10^4$). The average line width is measured for a local sampling of the microring (220 nm-347 nm), as depicted

in Suppl. Fig.16. The relationship between the two-photon absorption line width and the exposure dose is employed for fitting to obtain a consistency curve between the experiment and the fitting. Simultaneously, a 480 nm gap is achieved at N_{spp} = 7630 (2.83 fJ/(pulse·pixel)), and a minimum coupling gap exposure of 66 nm (Suppl. Fig.16) is attained at N_{spp} = 1.49 ×10^4 (5.72 fJ/(pulse·pixel)) and N_{pulse} = 2.1 × 10^7.

Supplementary Note 18: Comparison of resolution, power density, and throughput in different optical nanoprinting systems

We calculated the throughput values representing "efficiency" and provided a detailed comparison with similar methods and their relevant parameters (as shown in Suppl. Table 4). The Suppl. Table shows that our proposed method achieves high levels in both linewidth resolution and line spacing resolution, and its efficiency is on the same order of magnitude as similar methods, approximately 10^6 voxels/s. For traditional scanning-based techniques, achieving higher resolution requires smaller voxel volumes, which naturally reduces the area covered in a single exposure. As a result, printing the same area with higher resolution demands significantly more time. In contrast, the throughput of TPL remains largely unaffected by the number of voxels per layer due to its capability to simultaneously project an entire 2D layer (*16*). Our method employs a projection exposure system that efficiently balances resolution and throughput.

Supplementary Note 19: Attempts with different types of photoresists

Different types of materials were tested to study their compatibility with various lithography models, including SU-8, AR4340 (chemically amplified negative resist), AR5350 (non-chemically amplified positive resist), SCR500 (liquid radical resist), and silver/polymer nanocomposites. The diffusion introduced by the chemically amplified resist effectively enhanced the efficiency of TPDOPL, for example, the acid diffusion of AR5350 in post baking process led to one order lower the irradiated photon density comparing with that of the n-CA resist, AR-N 7520. The fabrication parameters and fabrication results are summarized as follows:

(1) SU-8 2000.5: The absorption spectrum of this negative photoresist shows no single-photon absorption above 400 nm (Suppl. Fig.17a). We have fabricated a hundred-micrometer structure using SU-8 at 305.92 μW, demonstrating its effectiveness for precision micro-structuring (Suppl. Fig.17 b).

(2) AR-N-4340: The absorption spectrum of this negative photoresist is below 400 nm (Suppl. Fig.17 c). In the experiment, a 1:2 diluted solution of the resist was used, and polymer lines with a width of 62 nm were successfully fabricated at power of 12.8 mW after the objective lens under the femtosecond laser irradiation of 517 nm (Suppl. Fig.17 d).

(3) SCR500: The absorption spectrum of this photoresist is below 500 nm (Suppl. Fig.17e). At a wavelength of 517 nm, a minimum polymer linewidth of 143 nm between square polymer structures was achieved using an average power of 33 mW after the objective lens (Suppl. Fig.17f).

(4) AR-N-5350: The absorption spectrum of this positive photoresist is below 500 nm (Suppl. Fig.17g). In the experiment, a grating with a width of 226 nm and a pitch of 376 nm was successfully fabricated using femtosecond laser irradiation at 517 nm, with a power of 1.5 mW after the objective lens (Suppl. Fig.17h).

(5) Silver/Polymer nanocomposite: In our precious work (Nano Lett. 2022, 22, 9823-9830), we have also explored the application of MOPL technique in the fabrication of functional nanocomposite structures using nanocomposite photoresist (Suppl. Fig.17l) under the femtosecond laser irradiation of 400 nm. The UV-Vis absorption spectra of 8.6×10^{-4} M aniline (ANI) and 6.0×10^{-3} M silver nitrate solution was measured. The results confirm that aniline shows absorption peaks at 230 nm and 280 nm, while silver nitrate displays absorption peak at 303 nm (Suppl. Fig.17m).

Supplementary information figures

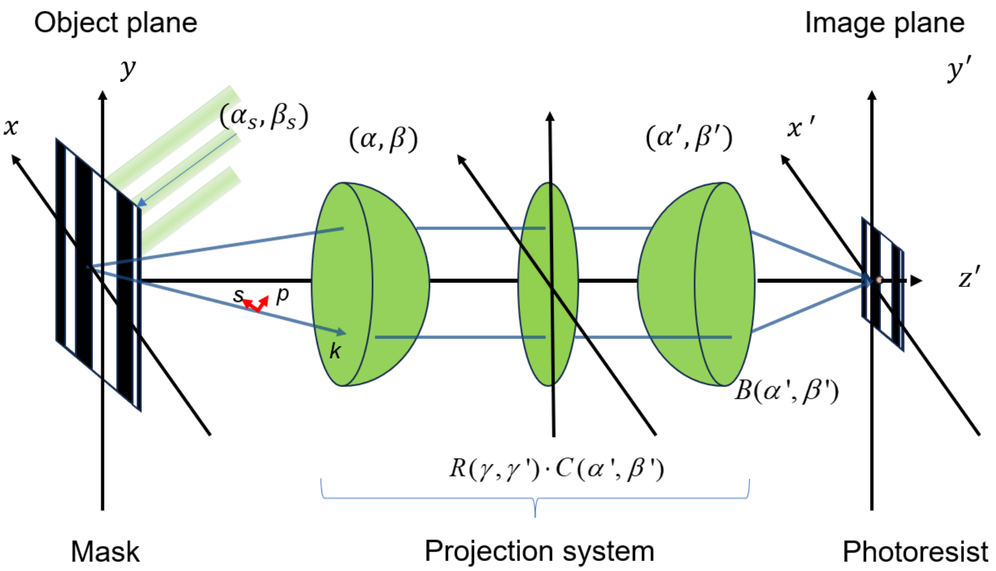

Suppl. Fig.1 Schematic diagram of digital-mask projection lithography. The illumination source is considered to be an axis point light source since the projection system uses parallel light with polarization direction in the x direction to uniformly illuminate the object surface where the DMD is located. The illumination light shines on the digital mask, and then modulates the light field on each corresponding pixel of the projection system projecting the light field of the object plane onto the image plane where the photoresist is located.

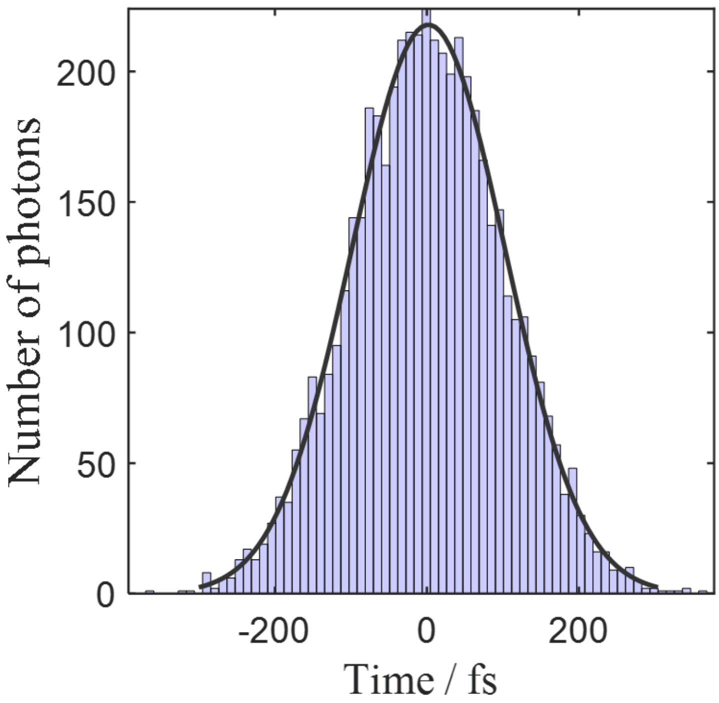

Suppl. Fig.2 Photon distribution within a single pulse (pulse width ~238 fs).

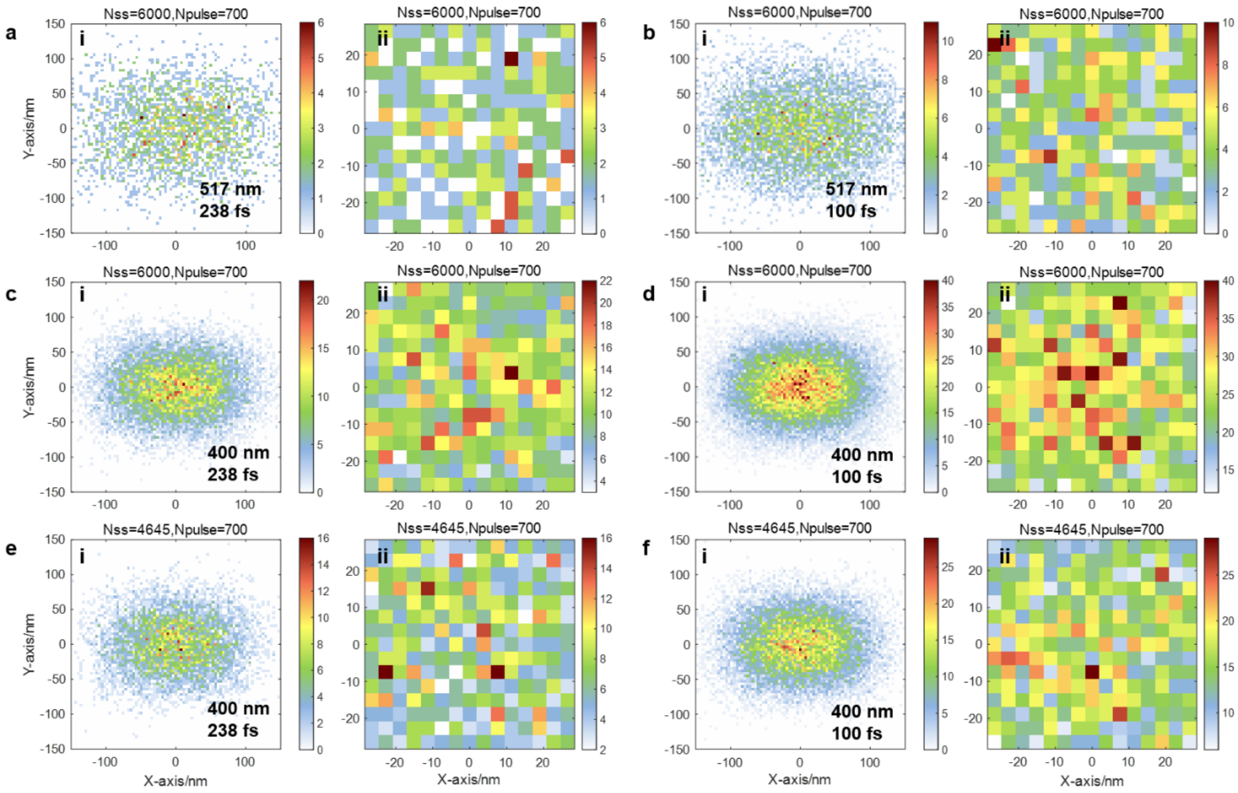

Suppl. Fig.3 The distribution of *e*TPA at different wavelengths and pulse widths. (a) λ = 517 nm, Γ= 238 fs. (b) λ = 517 nm, Γ= 100 fs. (c) λ = 400 nm, Γ= 238 fs. (d) λ = 400 nm, Γ= 100 fs. (e) λ = 400 nm, Γ= 238 fs, N_{spp} = 4645. (f) λ = 400 nm, Γ= 100 fs, N_{spp} = 4645. (ii) is the distribution at the center 50 nm^2 of (i).

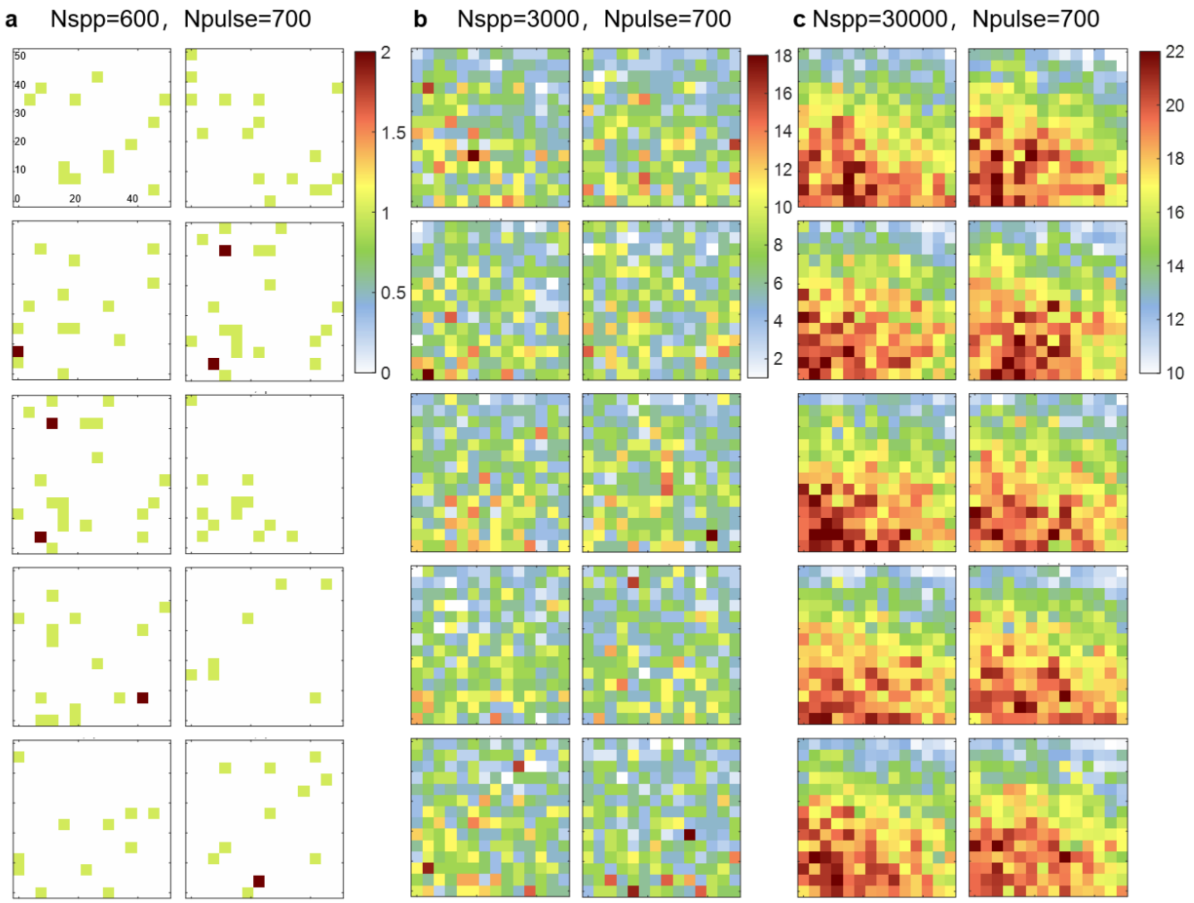

Suppl. Fig.4 Ten random distributions of TPA reaction probability in the 50 nm × 50 nm area at center of the spot under different injected photon numbers. (a) N_{spp} = 600, C.V = 26.38%. (b) N_{spp} = 6000, C.V = 2.76%. (c) N_{spp} = 30000, C.V = 0.65%.

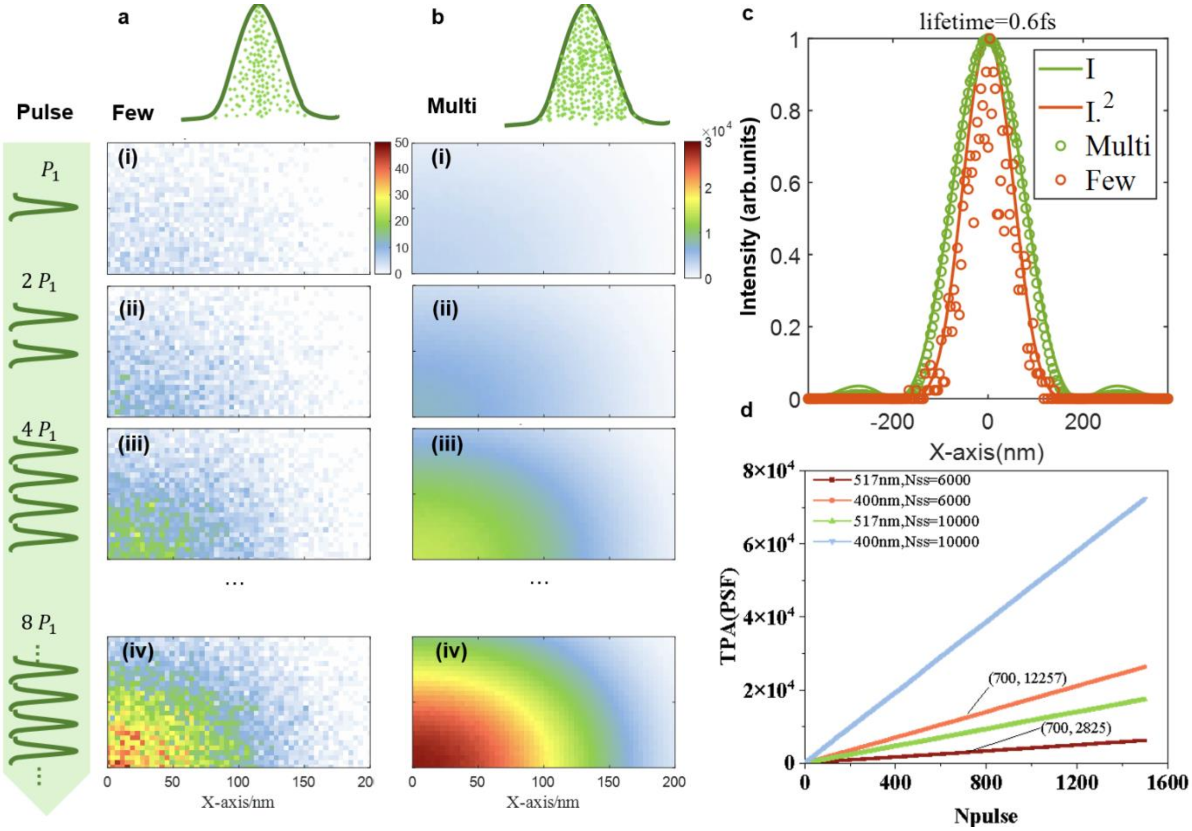

Suppl. Fig.5 TPA distribution of spot with different pulse. (a) TPA distribution of spot with an increased pulse under 517 nm few photons. (b) TPA distribution of spot with an increased pulse under 517 nm multi-photon absorption. (c) 517 nm multi-photon and few-photon spot cross-sectional distribution and the distribution of intensity and square of intensity. (d) The changing trend of spot TPA after increasing the number of pulses at different photon numbers at 400 nm and 517 nm.

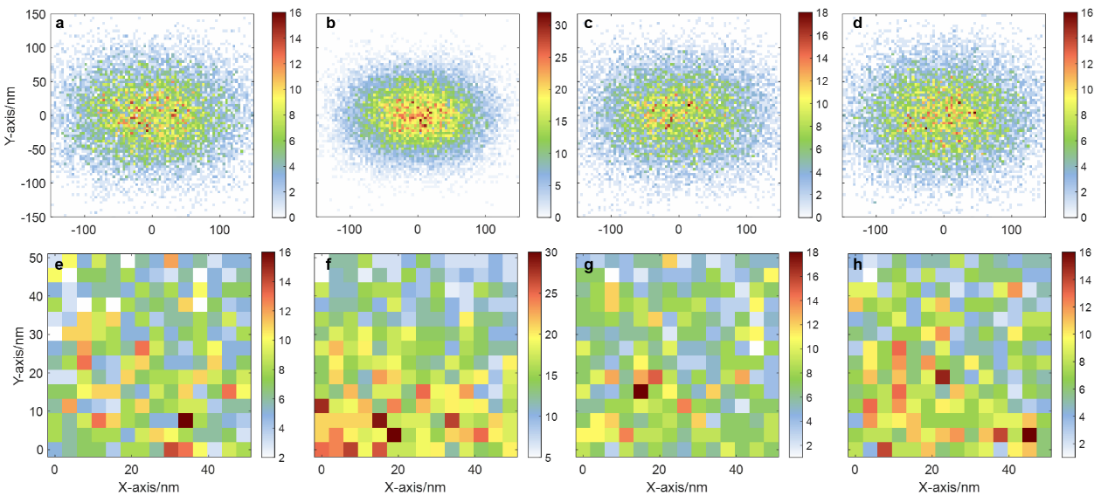

Suppl. Fig.6 TPA distribution of spot with different parameters. (a-d) The probability distribution of TPA in the laser spot under different parameters (N_{pulse} = 700, N_{spp} = 6000). (a) The original parameters of the experimental system, λ = 517 nm, FWHM = 238 fs, NA = 1.49. (b) λ = 400 nm, FWHM = 238 fs, NA = 1.49. (c) Reduce the laser pulse width to 100 fs. (d) λ = 517 nm, FWHM = 238 fs, NA = 1.45. (e-h) *e*TPA reaction corresponding to 50 nm^2 in the center of the spot under different parameter conditions.

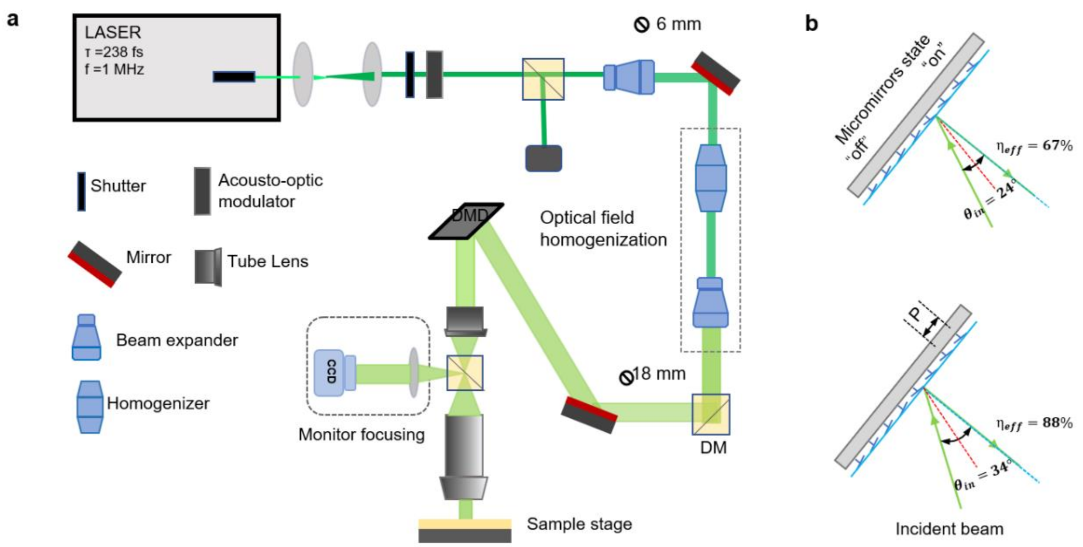

Suppl. Fig.7 Two-photon absorption projection optical system. (a) Schematic illustration of the experimental setup. (b) The effective energy utilization($\boldsymbol{\eta_{eff}}$) obtained at different incident angles ($\boldsymbol{\theta_{in}}$) ,the optimized incident angle is =34°(P = 7.56 μm).

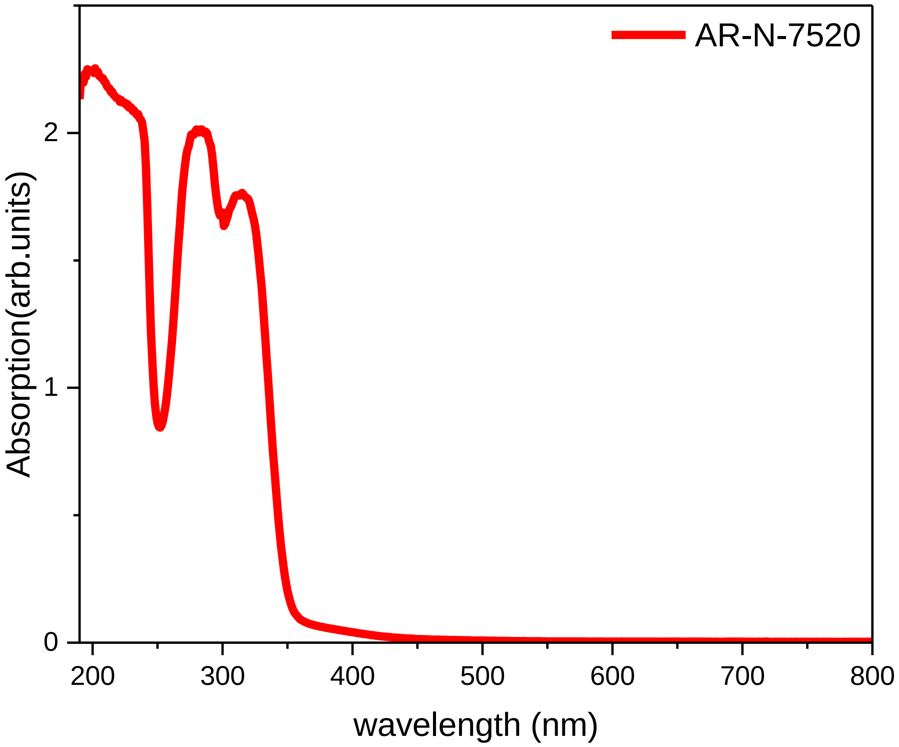

Suppl. Fig.8 UV-Vis absorption spectrum of AR-N-7520.

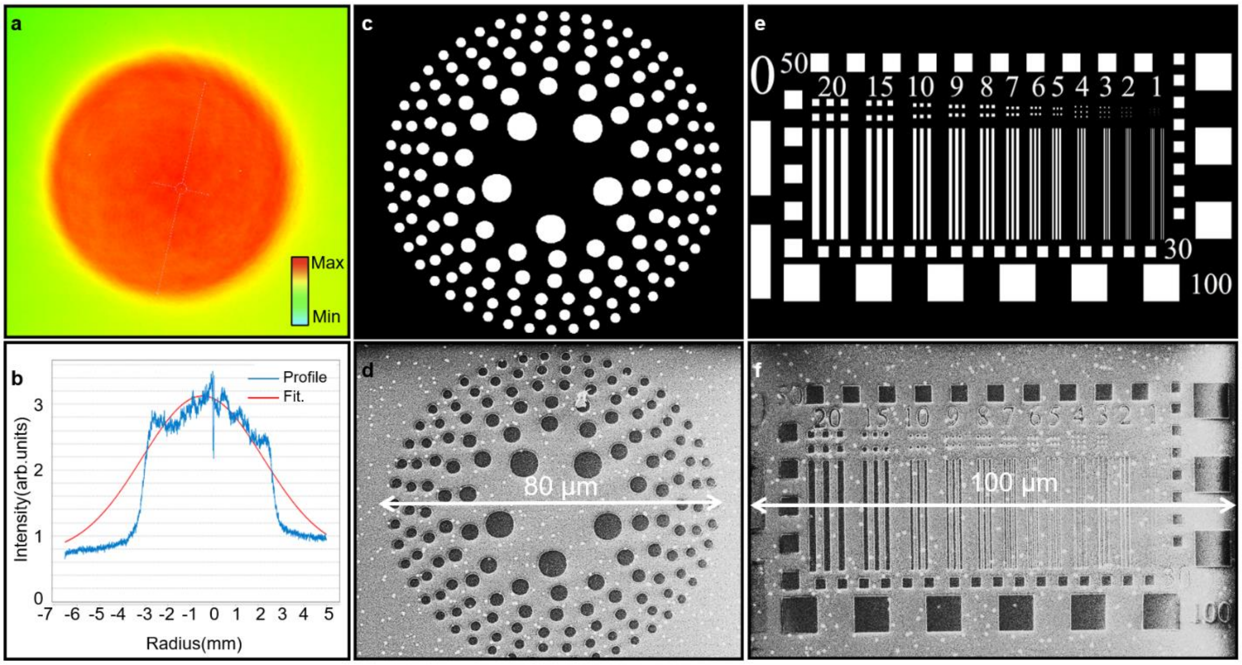

Suppl. Fig.9 Light intensity uniformity characterization. (a-b) Spot shape after homogenization (spot diameter 6.4 mm). (c-d) Design layout and experimental results of photon sieve (diameter 80 μm). (e-f) DMD layout of resolution plate and experimental SEM results (long side 100 μm).

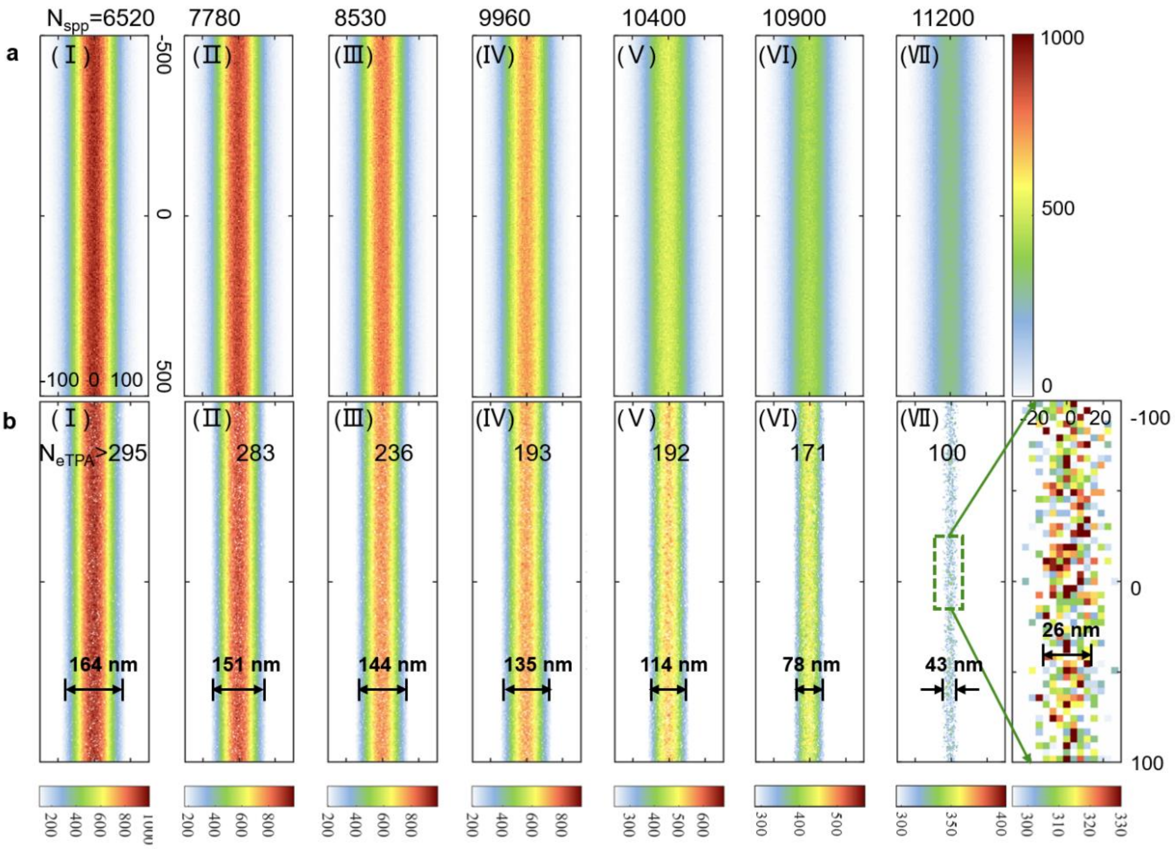

Suppl. Fig.10 Distribution of N_{eTPA} within a line of 1 pixel width under different N$_{spp}$. (a) N_{spp} is 6520(I), 7780(II), 8530(III), 9960(IV), 10400(V), 10900(VI), 11200(VII), the total distribution of N_{eTPA} within a line with width of 1 pixel when the N_{pulse} is 6000. (b) The contour map corresponds to the distribution of experimental line widths, and the inset is the N_{eTPA} distribution map corresponding to 26 nm at the N_{spp} = 6520.

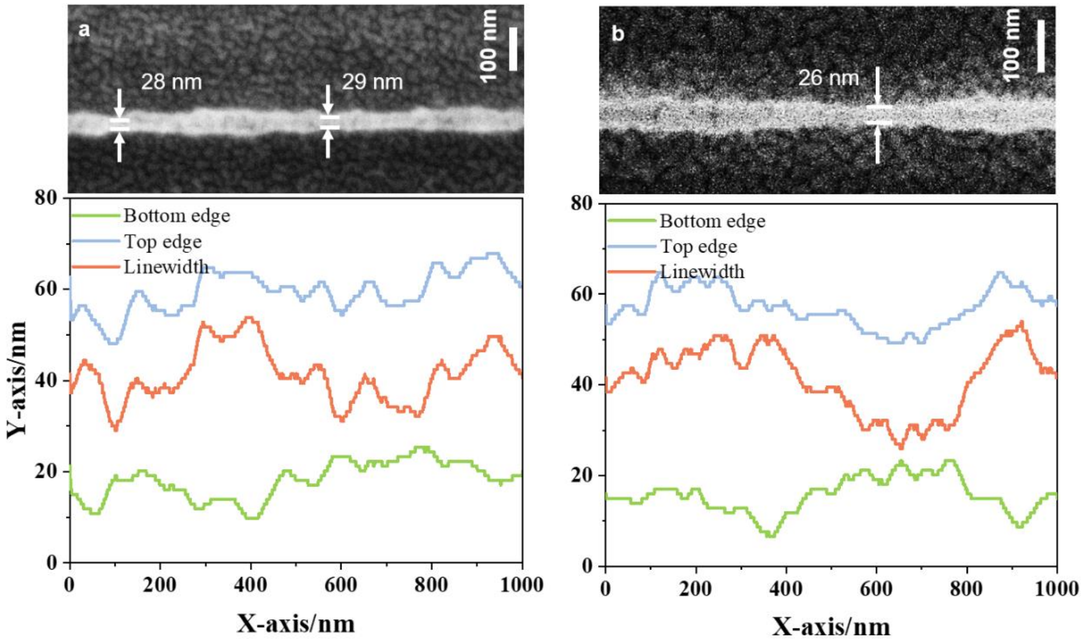

Suppl. Fig.11 Roughness with feature size. (a) Line edge roughness with a feature size of 28 nm. (b) Line edge roughness with a feature size of 26 nm.

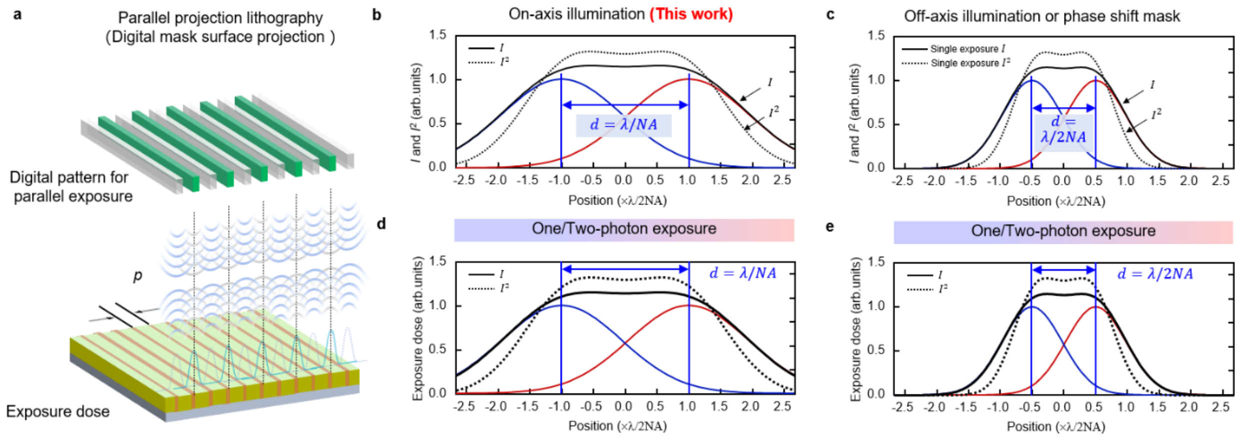

Suppl. Fig.12 Sparrow criterion for parallel projection lithography. (a) Schematic diagram of parallel projection lithography; (b, c) Calculated focal intensity distribution for single exposure under off-axis illumination and off-axis illumination (or phase shift mask), respectively; Lateral profiles of I (solid line) and I^2 (dotted line) correspond to one-photon exposure and two-photon exposure, respectively. (d, e) Critical lateral distance for one/two-photon single exposure under off-axis illumination and off-axis illumination (or phase shift mask), respectively. The red and blue lines represent the intensity distributions of the light field for the two nanolines, obtained from two separate scanning sessions.

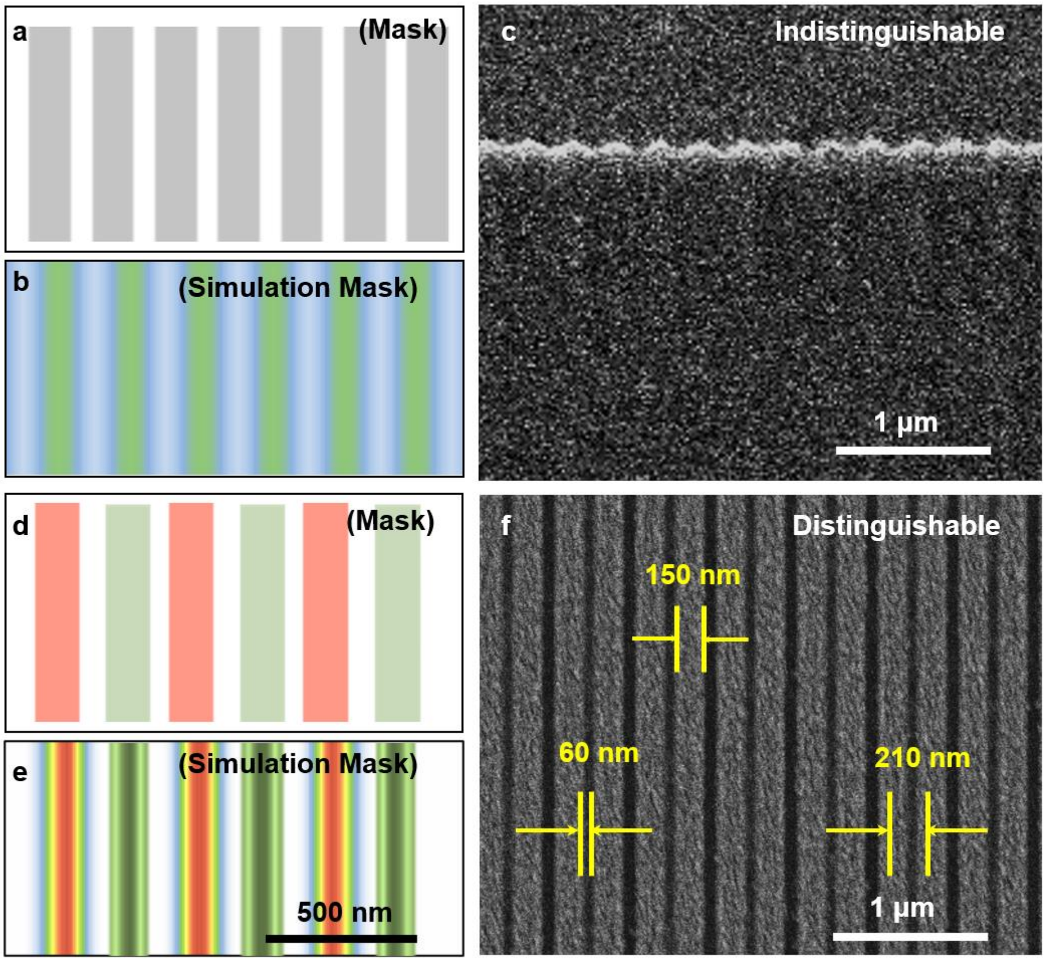

Suppl. Fig.13 Details of *i*DME processing. (a) Single exposure mask layout, duty cycle 2:1. (b) 2:1 grating single exposure mask calculated square distribution of light intensity. (c) 2:1 grating line group single exposure, indistinguishable. (d) In-situ double exposure layout, single exposure duty cycle 2:4, using two colors represent two exposures respectively. (e) 2:1 grating double exposure mask calculated square distribution of light intensity. (f) 2:1 grating line group single exposure, resolvable period 210 nm.

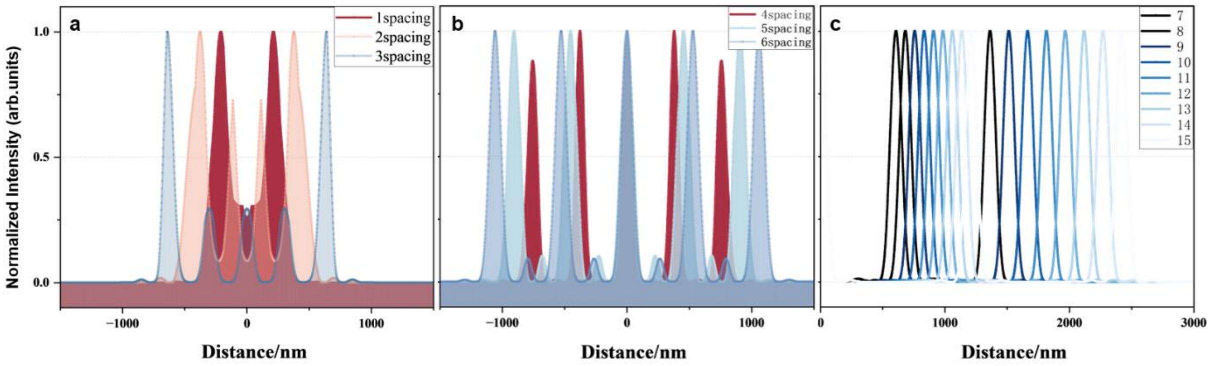

Suppl. Fig.14 Calculation and simulation results of light intensity of 5 lines with different intervals. (a) Line group with a spacing of 1-3 pixels, completely losing its original appearance. (b) Line group with a spacing of 4-6 pixels, uneven intensity distribution. (c) Line group with spacing of 7-15 pixels, appearance and intensity distribution is not affected by adjacent lines.

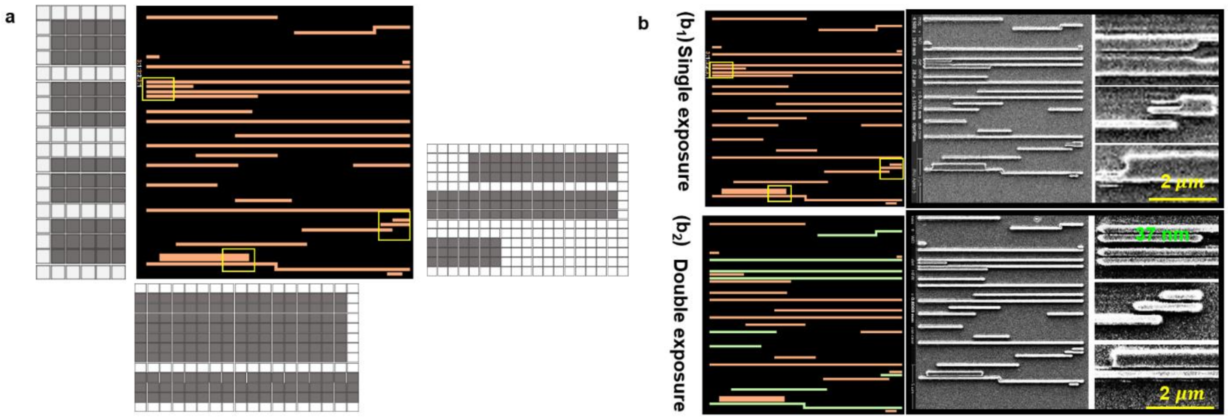

Suppl. Fig.15. Exposure results of the basic circuit layout. (a) Local 1-pixel enlarged view of the basic circuit layout of the metal layer. (b) SEM image of the exposure results with a minimum gap of 37 nm.

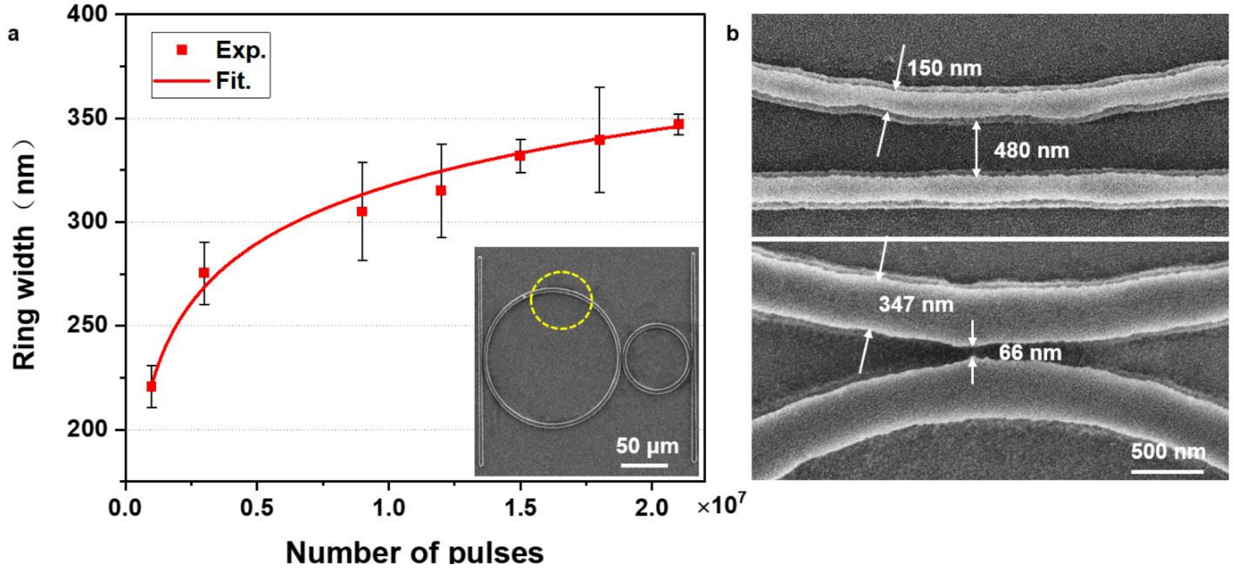

Suppl. Fig.16 Exposure results of the microring layout. (a) The changing trend of the line width of microring diameter with the increase of exposure time. (b) Exposure to different coupling gaps. Error bars represent mean ± SD based on 10 independent measurements for each data point.

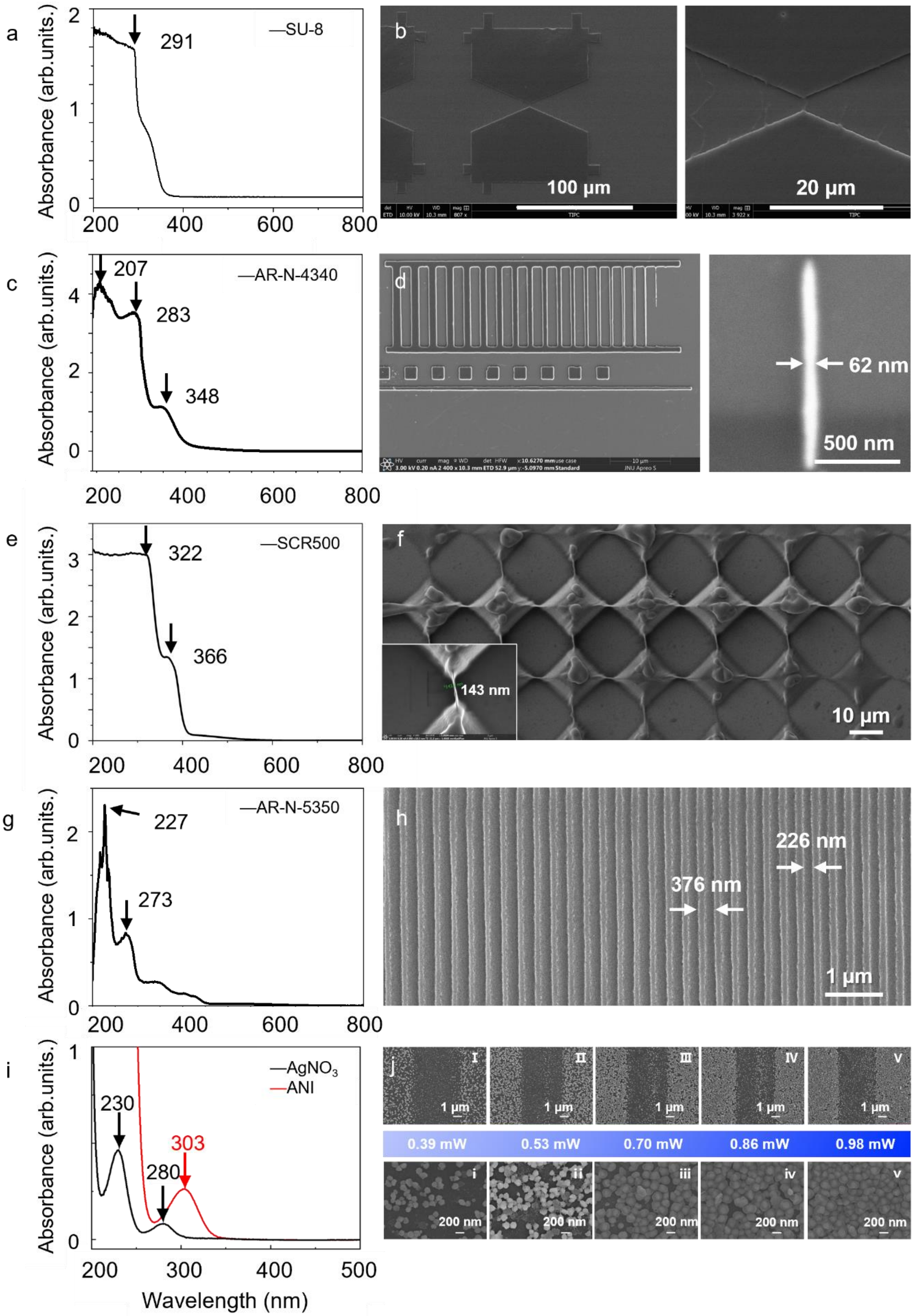
a
Absorbance (arb.units.)
291
—SU-8
b
100 μm
20 μm
c
207
283
348
—AR-N-4340
d
62 nm
500 nm
e
322
366
—SCR500
f
143 nm
10 μm
g
227
273
—AR-N-5350
h
226 nm
376 nm
1 μm
i
—AgNO3
—ANI
230
303
280
Wavelength (nm)
j
I
II
III
IV
V
1 μm
0.39 mW
0.53 mW
0.70 mW
0.86 mW
0.98 mW
i
ii
iii
iv
v
200 nm

Suppl. Fig.17 UV-Vis absorption spectra and exposure results of different photoresist systems. (a) UV-Vis absorption spectrum of SU-8 film; (b)Fabrication of a hundred-micrometer structure using photoresist SU-8; (c) Absorption spectrum of AR-N-4340; (d) Exposure of lines with varying pixel widths using photoresist AR-N-4340, achieving a minimum line width of 62 nm; (e) UV-Vis absorption spectra of SCR500; (f) Fabrication of a hundred-micrometer structure using photoresist SCR500, the illustration shows an SEM image of the minimum polymer linewidth of 143 nm; (g) Absorption spectrum of AR-N-5350; (h) The grating lines fabricated using SCR500 photoresist have a period of 376 nm and a trench of 226 nm.(i) UV-Vis absorption spectra of 8.6 $\times 10^{-4}$ M ANI and 6.0 $\times 10^{-3}$ M $AgNO_3$ solutions; (j) SEM images of Silver/Polyaniline nanocomposites prepared with different laser powers (0.39~0.98 mW) at low (I−V) and high magnification (i−v). The black arrows represent the absorption peaks.

Supplementary information tables

Suppl. Table 1 Numerical values of parameters in the optical model for few-photon absorption.

Parameter	Numerical	Numerical	Units
Cutoff Line	353	353	nm
Numerical Aperture (NA)	1.45	1.49	Non-dimensional
Wavelength (λ)	400	517	nm
Virtual life	0.7973	0.2953	fs
FWHM	100	238	fs
magnification	90	100	Non-dimensional
refractive_index (photoresist)	1.623	1.622	Non-dimensional
DMD_size	7.56	7.56	μm

Suppl. Table 2 The efficiency of TPA occurrence varies under different situations.

Wavelength	Pulse width	N_{spp}	N_{pulse}	N_{eTPA} (PSF)	N_{eTPA} (50×50 nm^2)	Efficiency of PSF (%)	Efficiency of 50×50 nm^2(%)
517	238	6000	700	2971	360	0.07	0.01
400	238	6000	700	12261	2481	0.29	0.06
517	100	6000	700	6970	857	0.17	0.02
400	100	6000	700	28716	5767	0.68	0.14
400	238	4645	700	7198	1488	0.22	0.05
400	100	4645	700	17146	3372	0.53	0.10

Suppl. Table 3 Analysis of the comprehensive dispersion degree of light spots after 50 random processes.

N_{spp}	N_{pulse}	$N_{eTPA}^{Ave.}$ of PSF	N_{eTPA}^{SD} of PSF	C_V of PSF	C_V of 50 nm^2 in the center of PSF
600	700	143	14	9.79%	26.38%
6000	700	14049	115	0.82%	2.76%
30000	700	340359	521	0.15%	0.65%

SD: Standard Deviation ；Ave.：Average Number.

Suppl. Table 4 Comparison of Resolution, Power Density, and Throughput in Different 3D Printing Techniques.

Year Group	Method		Source Power density	Resolution	Throughput	
	Princ iple	Type			Fabrication rate	Voxel fabricatio n rate (voxels/s)
2013 [15] Benjami n Mills	MPP	DMD projecti on	Femtosecond amplified laser, 800 nm,150 fs,1 kHz; Single pulse 1 mJ	Width: 650 nm (0.45λ/NA); Pitch: no data	2D: 0.5mm^2/min (8.3×10^{-3} mm^2/s) 3D: 4.5×10^{-3} mm^3/s (Thickness=20 μm)	No data
2019 [16] Shih-Chi Chen	TPP	DMD projecti on	Femtosecond amplified laser, 800 nm, 35fs, ~40 nm bandwidth, 1 kHz, P=~1W; 1 TW/cm^2	Lateral widths: 130-140 nm (~0.21λ/NA); Axial height: 175 nm Pitch: no data	2D: ~9 mm^2/s 3D: 5-20mm^3/hour (0.83-3.33)$\times10^{-2}$ mm^3/s	3.33×10^{8}
2021[17] Xianfan Xu	TPP	DMD projecti on+ spatiot empor al focusin g	Femtosecond amplified laser, 800 nm, 65 fs, 22 nm bandwidth; 5 kHz 0.44 TW/cm^2	Width: ~200 nm (0.37λ/NA); Layer thickness: ~1 μm Pitch: no data	2D: ~7.2×10^{-2}mm^2/s (12 μm × 12 μm) 3D: ~10^{-3} mm^3/s	3.2×10^{6}
2021[18] Xuan-Ming Duan	TPP	DMD projecti on	Femtosecond laser oscillator, 400 nm, 100 fs, 80 MHz, P=~1W; 0.14-0.365	Width: 32 nm (0.12λ/NA); Pitch: no data	2D: 1.74×10^{-2} mm^2/s 3D: 1.116×10^{-5} mm^3/s (Thickness=155 nm)	~1×10^{6}

			MW/cm^2			
2022 [19] Martin Wegener	TSA +LCD	Light-sheet	Four 6 W optical-power, 440 nm wavelength laser diodes I_1 = 162 μW /μm^2 (0.0162 MW/cm^2)	Width: 500 nm; Minimum ~250 nm (0.68λ/NA) Pitch: no data	2D:2.4×10^{-2} mm^2/s (suspended lines with a pitch of 6 μm and length of 20 μm) 3D:3.85×10^{-3} mm^3/s (3.85×10^6 μm^3/s)	7×10^6
2023 [20] Shih-Chi Chen	TPP	DMD Holography multi-foci	Femtosecond amplified laser, 800 nm,100fs,1kHz, P=4 W; 3.3~22.7 TW/cm^2	Width: 90 nm (0.15λ/NA); Pitch: ~500 nm (0.81λ/NA)	2D: 5.4×10^{-2} mm^2/s (9 μm×9 μm,150 ms) 3D: 1.5×10^{-3} mm^3/s (54 mm^3/hour)	2×10^6
2024 [21] Martin Wegener	TPP	DOE multi-foci (7 × 7)	Femtosecond laser oscillator, 790nm,140fs,80MHz, P=3.7W; 1.2TW/cm^2 per focus	Mean lateral size: 475 nm (0.84λ/NA) Pitch: no data	2D: 7×10^{-2} mm^2/s (2-inch wafers 8 hour) 3D: 3.6×10^{-3} mm^3/s	10^8
**This work**	**TPP**	**DMD projection**	**Fiber Femtosecond Laser, 517nm, ~240 fs, 1 MHz; 0.17 GW/cm^2**	**Minimum width: 26 nm (0.075λ/NA); Pitch: 210 nm (0.6λ/NA)**	**~1×10^{-3} mm^2/s(26 nm) ~0.1 mm^2/s (250 nm)**	**~4×10^6**

Supplementary references:

1. X. Yu Yan et al., The photon detection mode of photomultiplier tubes considering the pulse height distribution. *Journal of Infrared Millimeter Waves* 42, 88-101 (2023).
2. A. Laskin et al., in Components and Packaging for Laser Systems. (*SPIE*, 2015), vol. 9346, pp. 224-232
3. M. J. Deng, Y. Y. Zhao, Z. X. Liang, J. T. Chen, Y. Zhang, X. M. Duan, Maximizing energy utilization in DMD-based projection lithography. *Opt. Express* 30, 4692–4705 (2022).
4. TWINSCAN NXE:3400C – EUV lithography systems | ASML https://www.asml.com/en/products/euv-lithography-systems/twinscan-nxe3400c
5. Kang M, Han C, Jeon H. Submicrometer-scale pattern generation via maskless digital photolithography[J]. *Optica*, 7(12): 1788-1795 (2020).
6. Somers P, Koch S, Kiefer P, et al. Holographic multi-photon 3D laser nanoprinting–at the speed of light: opinion[J]. *Optical Materials Express*, 14(10): 2370-2376 (2024).
7. Ge Q, Li Z, Wang Z, et al. Projection micro stereolithography based 3D printing and its applications[J]. *International Journal of Extreme Manufacturing*, 2(2): 022004(2020).
8. A. Erdmann, Optical and EUV Lithography: A Modeling Perspective. *SPIE* (2021).
9. F. M. Schellenberg, Resolution enhancement technology: the past, the present, and extensions for the future. *Proc. SPIE* 5377, Optical Microlithography XVII, 1–20 (2004).
10. J. Fischer, M. Wegener, Three-dimensional optical laser lithography beyond the diffraction limit. *Laser Photonics Rev*. 7, 22–44 (2013).
11. C. M. Sparrow, On spectroscopic resolving power. *Astrophys. J.* 44, 76 (1916).
12. B. R. Masters, Abbe’s theory of image formation in the microscope. Superresolution Optical Microscopy: The Quest for Enhanced Resolution and Contrast, 65–108 (2020).
13. M. Shibuya, Resolution enhancement techniques for optical lithography and optical imaging theory. *Opt. Rev.* 4, 151–160 (1997).
14. M. D. Levenson, N. S. Viswanathan, R. A. Simpson, Improving resolution in photolithography with a phase-shifting mask. IEEE Trans. Electron Devices 29, 1828–1836 (1982).
15. B. Mills, J. A. Grant-Jacob, M. Feinaeugle, R. W. Eason, Single-pulse multiphoton polymerization of complex structures using a digital multimirror device. *Opt. Express* 21, 14853–14858 (2013).
16. S. K. Saha, D. Wang, V. H. Nguyen, Y. Chang, J. S. Oakdale, S.-C. Chen, Scalable submicrometer additive manufacturing. *Science* 366, 105–109 (2019).
17. P. Somers, Z. Liang, J. E. Johnson, B. W. Boudouris, L. Pan, X. Xu, Rapid, continuous projection multi-photon 3D printing enabled by spatiotemporal focusing of femtosecond pulses. *Light Sci. Appl*. 10, 199 (2021).

18. Y.-H. Liu, Y.-Y. Zhao, F. Jin, X.-Z. Dong, M.-L. Zheng, Z.-S. Zhao, X.-M. Duan, λ/12 super resolution achieved in maskless optical projection nanolithography for efficient cross-scale patterning. *Nano Lett*. 21, 3915–3921 (2021).
19. V. Hahn, P. Rietz, F. Hermann, P. Müller, C. Barner-Kowollik, T. Schlöder, W. Wenzel, E. Blasco, M. Wegener, Light-sheet 3D microprinting via two-colour two-step absorption. *Nat. Photonics* 16, 784–791 (2022).
20. W. Ouyang, X. Xu, W. Lu, N. Zhao, F. Han, S.-C. Chen, Ultrafast 3D nanofabrication via digital holography. *Nat. Commun*. 14, 1716 (2023).
21. P. Kiefer, V. Hahn, S. Kalt, Q. Sun, Y. M. Eggeler, M. Wegener, A multi-photon (7 × 7)-focus 3D laser printer based on a 3D-printed diffractive optical element and a 3D-printed multi-lens array. *Light Adv. Manuf*. 4, 28–41 (2024).
